# Preference index supported by motivation tests in Nile tilapia

**DOI:** 10.1371/journal.pone.0175821

**Published:** 2017-04-20

**Authors:** Caroline Marques Maia, Gilson Luiz Volpato

**Affiliations:** Laboratory of Animal Physiology and Behavior, Institute of Biosciences (IB), UNESP, Botucatu, São Paulo, Brazil; Radboud University Medical Centre, NETHERLANDS

## Abstract

The identification of animal preferences is assumed to provide better rearing environments for the animals in question. Preference tests focus on the frequency of approaches or the time an animal spends in proximity to each item of the investigated resource during a multiple-choice trial. Recently, a preference index (PI) was proposed to differentiate animal preferences from momentary responses (Sci Rep, 2016, 6:28328, DOI: 10.1038/srep28328). This index also quantifies the degree of preference for each item. Each choice response is also weighted, with the most recent responses weighted more heavily, but the index includes the entire bank of tests, and thus represents a history-based approach. In this study, we compared this PI to motivation tests, which consider how much effort is expended to access a resource. We performed choice tests over 7 consecutive days for 34 Nile tilapia fish that presented with different colored compartments in each test. We first detected the preferred and non-preferred colors of each fish using the PI and then tested their motivation to reach these compartments. We found that fish preferences varied individually, but the results were consistent with the motivation profiles, as individual fish were more motivated (the number of touches made on transparent, hinged doors that prevented access to the resource) to access their preferred items. On average, most of the 34 fish avoided the color yellow and showed less motivation to reach yellow and red colors. The fish also exhibited greater motivation to access blue and green colors (the most preferred colors). These results corroborate the PI as a reliable tool for the identification of animal preferences. We recommend this index to animal keepers and researchers to identify an animal’s preferred conditions.

## Introduction

To better identify resources for environmental enrichment in captivity, some authors have proposed evaluating an animal’s preferences. The detection of such preferences is usually based on multiple-choice decisions (e.g. [1, 2]). This approach assumes that what is chosen in the test is preferred by the animal and should be used in its captive environment. However, does a momentary choice represent the consistent preference of the animal?

This issue was recently investigated and the use of a Preference Index (PI) was proposed [3]. The study showed that choices can indicate momentary (non-preference) or consistent (preference), more long-term responses and focused on a tool (the PI) to differentiate preferred from non-preferred choice items. The Preference Index is a history-based method that considers the entire history of choices made by the animal over a period of time such that the more recent the choice, the greater its weight in the calculations; this is done because recent choices should better represent the actual condition of the animal [3]. However, less recent choices still have a role in the PI because they are an expression of the animal’s choice at a previous time. Thus, the PI weights these responses and provides a clear-cut graph to differentiate between the occurrence of preferred and non-preferred items. Moreover, as more choice tests are inserted into the calculation, the Preference Index is less affected by the results of a few momentary tests [3]. In addition to the qualitative detection of preferences and non-preferences, the index quantifies the extent of these responses, thus detecting the intensity of each preferred or non-preferred response.

Although the PI is a promising method for detecting animal preferences to improve the quality of conditions for animals in captivity, additional research is still needed. A necessary step to validate the PI is to compare this index to motivation tests. Such tests for motivation (e.g. [4–8]), measured in terms of how much effort the animal spends to reach a goal, can identify the importance of specific environmental resource items for the animals. Preference and motivation tests help to identify factors that contribute to the welfare of animals and are an improvement on assays that use physiological or other behavioral indicators of welfare, which are often challenging to interpret unequivocally [9–11].

As suggested by Duncan [12], the analysis of motivation should be used to complement the findings of preference tests. Detecting association between motivation and preference tests is also important because preference is more easily measured than motivation, although they have a different basis. For example, the PI indicates what item an animal prefers or does not prefer, whereas motivation indicates how much an item is preferred. Although the PI also quantifies the degree of preference [3], it quantifies how much an animal desires access to a resource and the motivation test indicates how much effort the animal is willing to expend to obtain it.

In this context, although a direct association between preference and motivation responses is expected, this relationship still needs to be demonstrated because preferred items and the motivation to access resources have been evaluated separately and for different resources. For example, although there are many studies evaluating the mating preferences of fish [13–15], to our knowledge, no study contrasted preference and motivation for this resource and only one study evaluated the fish motivation for a social partner [16]. Moreover, most preference or motivation studies have ignored individual variability, even though considerable individual variation in preference is known to exist [17–20], even when tests are conducted over several days [3].

Thus, we tested whether the determination of preference or non-preference items (via a PI) and the quantification of such preferences/non-preferences corresponded to the motivation of the same animals presented with the same resource items for each test. This allowed us to confirm the biological importance of Maia and Volpato’s PI [3] and emphasized the importance of individual variability in preference and motivation within an animal welfare context.

We focused on choice tests using Nile tilapia, *Oreochromis niloticus*, presented with different background colors. Nile tilapia are able to see colors, as this fish species has 7 cone opsin genes that exhibit distinct spectral sensitivities covering the entire visible spectrum, thus enabling the animal to see both shortwave and longwave colors [21]. Moreover, environmental color appears to be important to fish, as some colors have been demonstrated to influence the physiology and behavior of Nile tilapia and other fish species affecting, for example, the stress response [22–24], growth [22, 25–27], reproduction [28] and feeding [29, 30].

## Methods

### Animals and holding conditions

Adult Nile tilapia (*Oreochromis niloticus*) from a monoculture hatchery were held in indoor tanks (1000 to 1700 L) at a maximum density of 1 fish/5 l. The internal color of the tanks was grey, a color not tested in this study, thus minimizing any bias with regard to previous experience with environmental colors. The water quality was maintained at a temperature of 23.0 to 28.5°C, pH ≅ 7.0, ammonium < 0.3 ppm, and nitrite < 0.05 ppm. The photoperiod was 0600 h to 1800 h under white light; food (36% crude protein) was offered once a day (approximately 5% of biomass). All tanks were equipped with biological filters, constant aeration and a water circulation system. The fish remained in these holding conditions for at least 1 month prior to experimentation.

### Experimental design

We evaluated the association between preference and motivation responses for the same environmental resource. To accomplish this, we first evaluated the momentary choice responses of isolated fish (n = 34 fish) for background colors over 10 consecutive days to calculate their individual PI values (based on Maia & Volpato [3]). Individual fish were able to swim between four colored compartments in each daily test, and the fish position in the compartments was recorded every 30 s for 1 h/day during the last 7 days (test days); the first 3 days were used as an acclimation period to the experimental conditions. The PIs calculated from these choice frequencies indicated the preferred and non-preferred colors and the intensity of these preferences for each fish. On the 11^th^ day, a 5 min assay was used to test the motivation responses of the same fish to the colored compartments by recording the frequency of touches a fish gave to transparent, hinged doors that prevented access to each colored compartment.

### Measurements

**Fig 1:**
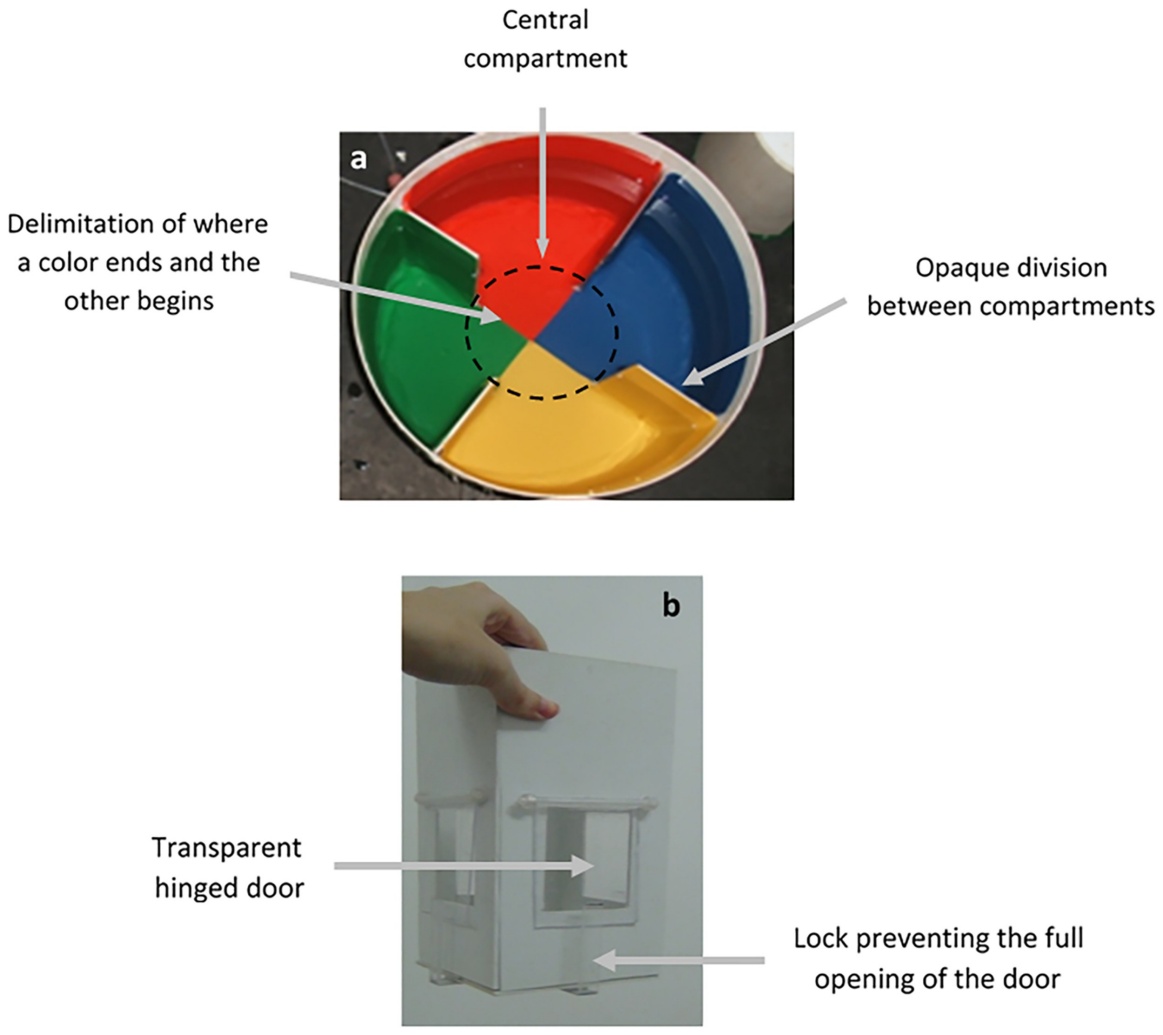
Overhead view of a circular test aquarium showing the four compartments with the same volume and area and the white, opaque chamber used in the motivation test. A) Each compartment was covered on the inside with adhesive paper of a different color (red, yellow, green or blue). The central compartment was formed by the junction of the background colors of the other compartments. The aquarium diameter was 40 cm; the water column height was 15 cm. The outer walls and the internal partitions were opaque and of the same color as the rest of the compartment. The test aquaria were randomly distributed within the testing area of the lab and the position of the colored compartments was changed for each fish to minimize any possible environmental bias in our results. B) Transparent locks attached to the bottom of the chamber prevented the complete opening of the transparent doors. Thus, fish could touch and move the doors, but could not completely open them and leave the central chamber. Chamber size = 10 x 10 x 22 cm.

After being measured (mean ± SD: 7.37 ± 0.17 cm, standard body length) and weighed, (12.08 ± 1.11 g), the fish were isolated (0800 h to 0900 h) in glass aquaria (40 x 20 x 25 cm) equipped with mechanical and chemical filters. Twenty-four hours later, we began preference tests only for the fish with clearer eyes, which indicates a low level or lack of stress [31]; this technique has been recently adapted for Nile tilapia [32]. Individual fish were placed in the central compartment of a circular aquarium (Fig 1A) divided into four compartments of the same size and of similar structure, but of different colors: red, yellow, green or blue. We chose to test these colors because some studies have shown an effect of these colors on the physiology and behavior of Nile tilapia [24, 27, 29, 30]; yellow was also identified as an apparent preferred color in this species [33].

Each fish was placed inside a 10 cm diameter transparent cylinder that was placed in the center of each circular aquarium (central compartment, Fig 1A). After 5 min, we carefully removed the cylinder, allowing fish to swim freely over the four compartments for 1 h while they were filmed from above. We then returned the fish to their respective isolation aquaria, where they were fed and remained until the next day’s test. This procedure was repeated for 10 consecutive days, but fish were filmed only on the last 7 days (test days). From the film, we recorded the position of the fish with respect to the colored compartments every 30 s for a total of 120 observation points during each daily test.

On the morning of the 11^th^ day, we tested the motivation of the fish to reach the different colored compartments using an opaque, white chamber (Fig 1B) in the center of each circular aquarium. Each chamber had four transparent hinged doors that faced one of the four colored compartments. An individual fish was placed inside the chamber and filmed from above for 5 min (a sufficient period to motivate the fish to touch on the doors, based on pilot tests). From the video analyses, we recorded the number of touches on each transparent door.

During all experimental procedures, the mechanical and chemical filters were kept in place in the isolation aquaria; we siphoned water and performed partial water changes in the circular test aquaria twice a week, always after the morning tests. This procedure maintained water quality at levels similar to those of the holding conditions. A white light (approximately 150 Lx) was placed approximately 2.5 m above the test aquaria. The temperature varied from 24.5°C to 29.0°C with the temperature variation distributed uniformly across all test aquaria. All fish were tested in the morning, as Nile tilapia were found to be more decisive (with regard to substratum resources) during this period of the day [34].

### Preference Index (PI) calculations

We calculated the PI values to determine the individual preference and non-preference responses as described in Maia and Volpato [3]. We calculated the cumulative frequency of fish positions over the test days and based on these data, we determined the cumulative area above the cumulative-frequency line. According to Maia and Volpato [3], the calculated area increases with the value of frequencies obtained in the most recent tests based on the assumption that the most recent choices may have a higher impact on the calculations, as the preference responses may vary over time. Calculations were made for each color option and for each individual fish. Positive PIs represent preferences and negative PIs represent non-preferences, and each PI value indicates the intensity of the response, thus identifying the most preferred and the most non-preferred (most avoided) colors for each fish (for details, see Maia and Volpato [3]).

### Statistical analyses

We compared the number of preferences for each color (how many times each color was preferred) among all fish (n = 34) and also for fish with only one preferred color (single-preference fish, n = 11) using Goodman’s proportion test [35]. Although single-preference fish were individuals that preferred just one color, all of these fish were assumed to be aware of the other available color options, as there was no case of a single-preference fish remaining in the same color during the entire observation time on all test days. Thus, all fish that expressed just one preference were included in the analyses. In the comparison of the PI values for different colors, we discarded data from 8 fish because there were problems with the videos of their preference tests. As the data of the remaining fish (n = 26), including the data of single-preference fish (n = 7), were not homogeneously distributed (Levene’s test, P < 0.05), we used a Friedman’s test for the comparisons among color. For the motivation test data, we used proportional data on the frequency of touches because the within-fish variation for this response was high. Thus, we compared the proportion of touches among the different colors using the data from all of the tested fish, including the data from the single-preference fish (n = 33 and 10, respectively; one fish did not touch at all). As these data were not normally distributed (Kolmogorov-Smirnov test, P < 0.05) and were not homogeneously variable (Levene’s test, P < 0.05), we used a Friedman’s test for these comparisons.

To determine whether individual fish were more motivated to access their own preferred colors, we compared the proportion of touches between the most preferred and the most avoided color for each individual (n = 33) using a Wilcoxon test, as these data were not normally nor homogeneously distributed (Kolmogorov-Smirnov and Levene’s tests, P < 0.05, respectively). We also compared the proportion of touches between all preferred and all non-preferred colors for each fish (n = 33) using a dependent t-test, as these data were normally and homogeneously distributed (Kolmogorov-Smirnov and Levene’s tests, P > 0.05, respectively). For all comparisons, we set α = 0.05.

### Ethical note

All experimental procedures were in accordance with the Ethical Principles for Animal Experimentation adopted by the Brazilian College of Animal Experimentation (COBEA) and were approved by the Ethics Committee on the Use of Animals (CEUA) at the Biosciences Institute of UNESP, Botucatu campus (SP-Brazil) (Protocol # 391-CEUA).

## Results

### Color preferences

**Fig 2:**
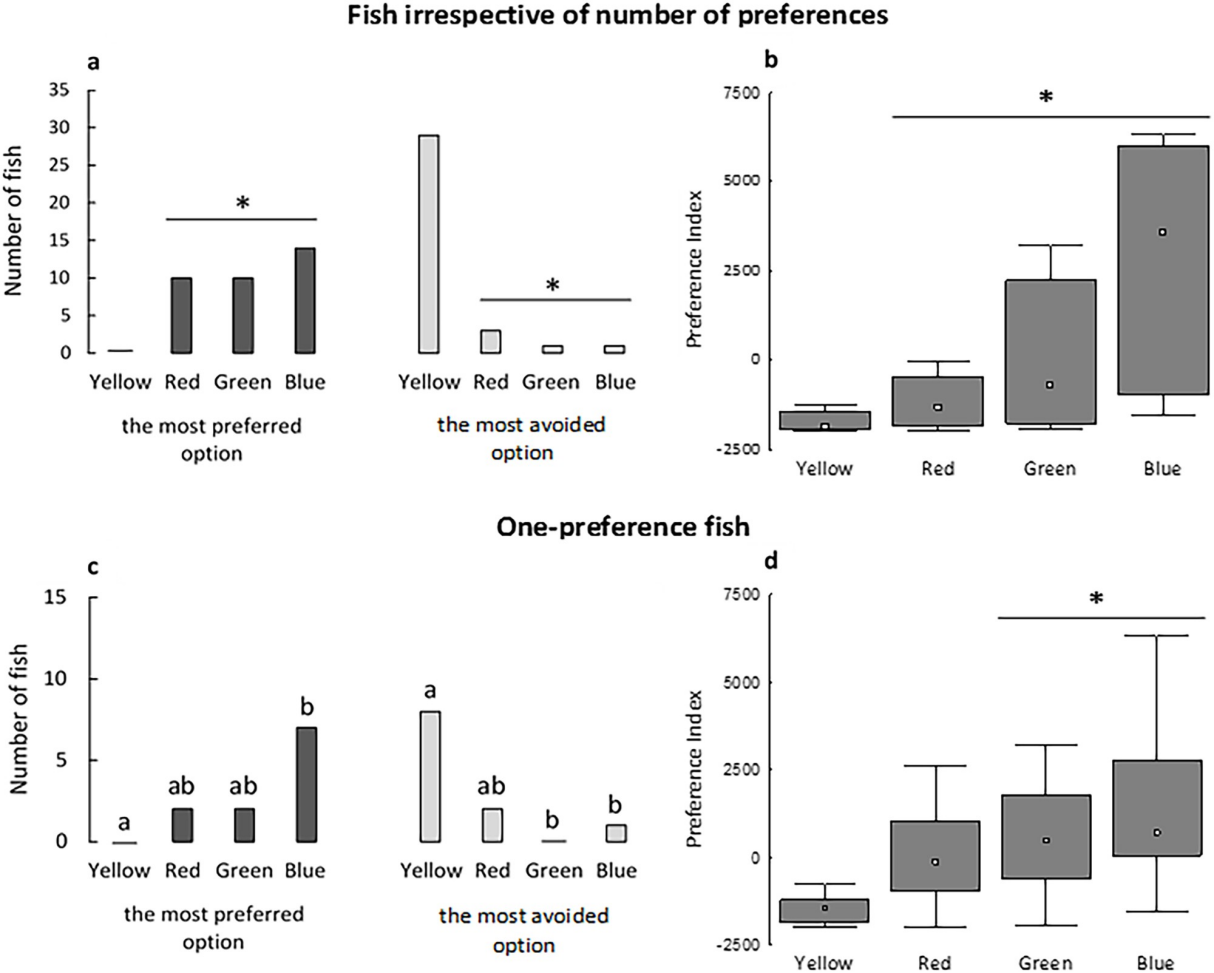
Background colors selected by fish as their most preferred or most avoided options, based on both the frequency of preference responses and the PI values for all tested fish (a and b, respectively) and for fish that preferred only one color (c and d, respectively). In B and D, box-plot: ^□^ indicates the median value; columns show the quartiles (Q1 and Q3) and bars indicate the maximum values. An asterisk (*) indicates a significant difference between colors in A (Goodman’s proportion test, P < 0.05), B (Friedman test, P < 0.0001, Fr = 37.06) and D (Friedman test, P < 0.007, Fr = 12.09). Numbers of fish with different lowercase letters are significantly different in C (Goodman’s proportion test, P < 0.05). N = 34 in A and C, n = 26 in B, n = 7 in D.

Among all tested fish, blue, green and red colors were identified as the most preferred colors with the highest PI median values (Fig 2A and 2B). In contrast, yellow was the most avoided (non-preferred) color (Fig 2A and 2B). We detected a similar trend when only single-preference fish were considered (Fig 2C and 2D). However, red and green were more similar to yellow when considering the most preferred color responses (Fig 2C), and red was more similar to yellow for both the most avoided color responses and the PI values (Fig 2D).

### Motivation for colors

**Fig 3:**
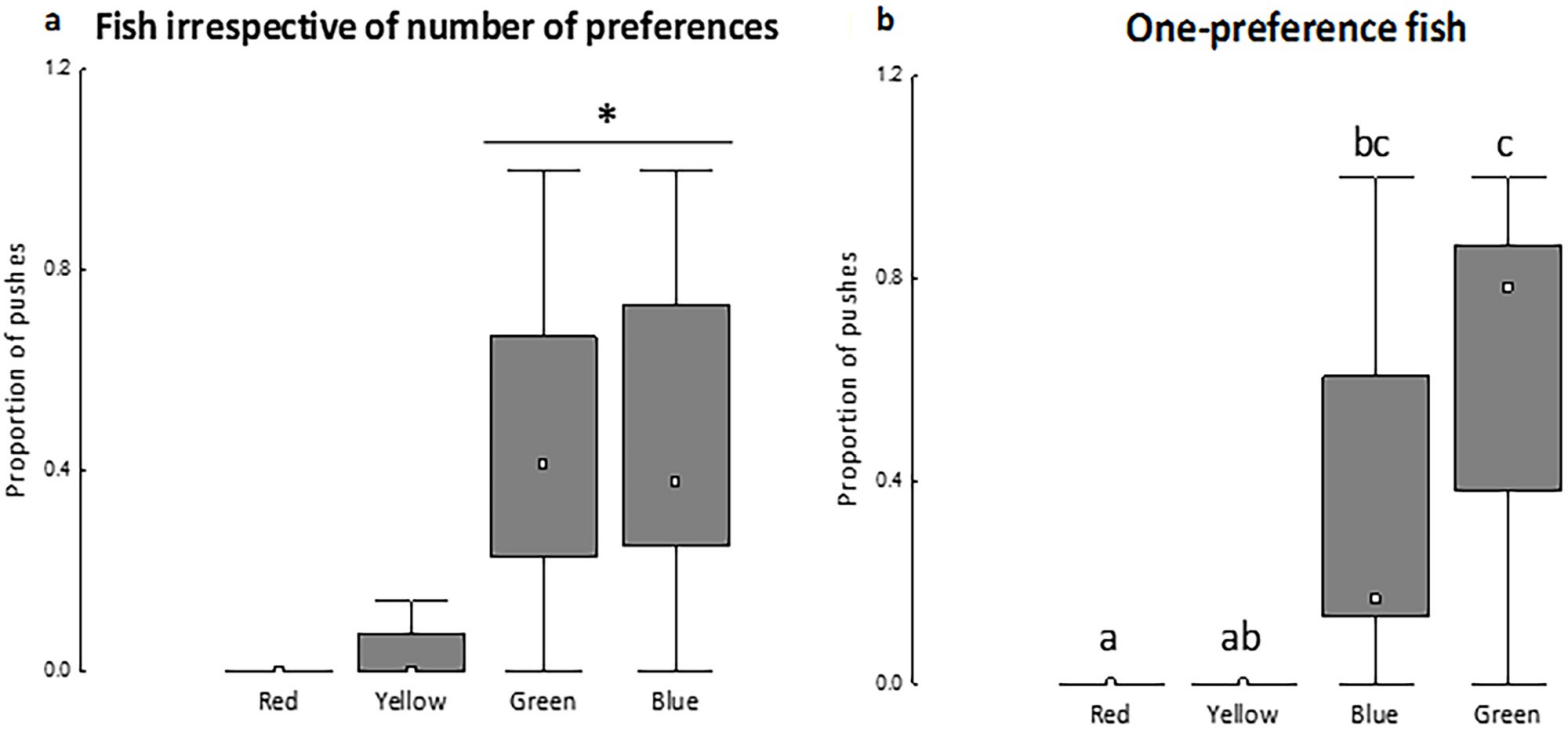
Proportion of touches on the transparent, hinged doors to each background color for all tested fish (a) and for fish that preferred only one color (b). Box-plot: ^□^ indicates the median value; columns show the quartiles (Q1 and Q3) and bars indicate the maximum values. In A, an asterisk (*) indicates a significant difference between colors (Friedman test, P < 0.0001, Fr = 61.47). In B, the proportion of touches with different lowercase letters are significantly different (Friedman test, P < 0.0003, Fr = 18.95). N = 33 in A, n = 10 in B.

All tested fish, except one, touched on the hinged doors during the 5 min trial of the motivation test (n = 33). Fish, regardless of the number of preference responses, more often touched the access doors to green and blue colors than to red and yellow colors (Fig 3A). Single-preference fish exhibited the same pattern, but with blue being more similar to yellow in the number of touches (Fig 3B).

### Motivation for individually preferred colors

**Fig 4:**
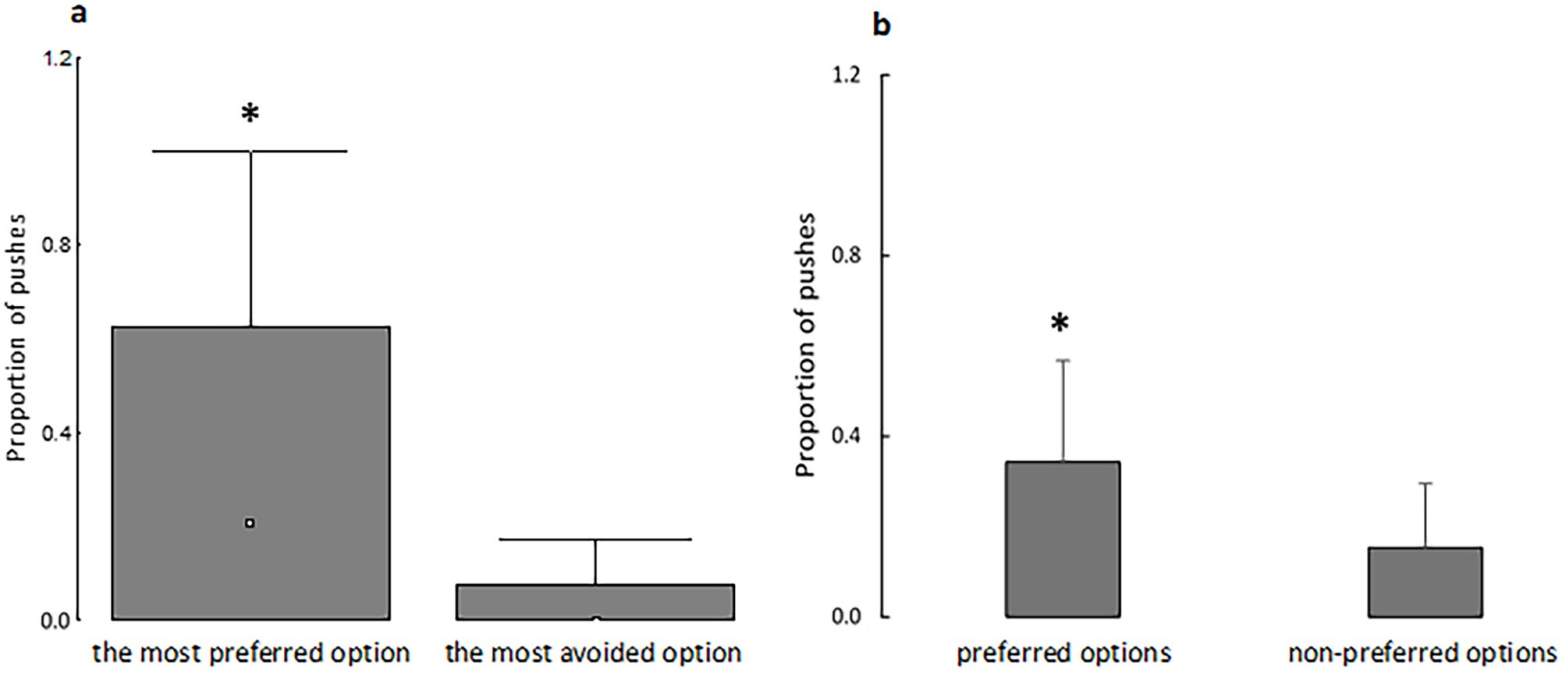
Proportion of touches made on the transparent, hinged doors to reach the individually most preferred and most avoided colors (a) or to access all of the individually preferred and non-preferred colors (b). In A, box-plot: ^□^ indicates the median value; columns show the quartiles (Q1 and Q3) and bars indicate the maximum value. In B (mean + SD), an asterisk (*) indicates a significant difference (Wilcoxon test, P = 0.002, Z = 3.03; dependent t-test, P = 0.003, t = 3.16). N = 33 in A and B.

Regardless of color, Nile tilapia more frequently touched to access their individually most preferred item, and touched less frequently for their individually most avoided item (Fig 4A). This same pattern was observed when all the preferred or the non-preferred items were considered (Fig 4B).

**Fig 5:**
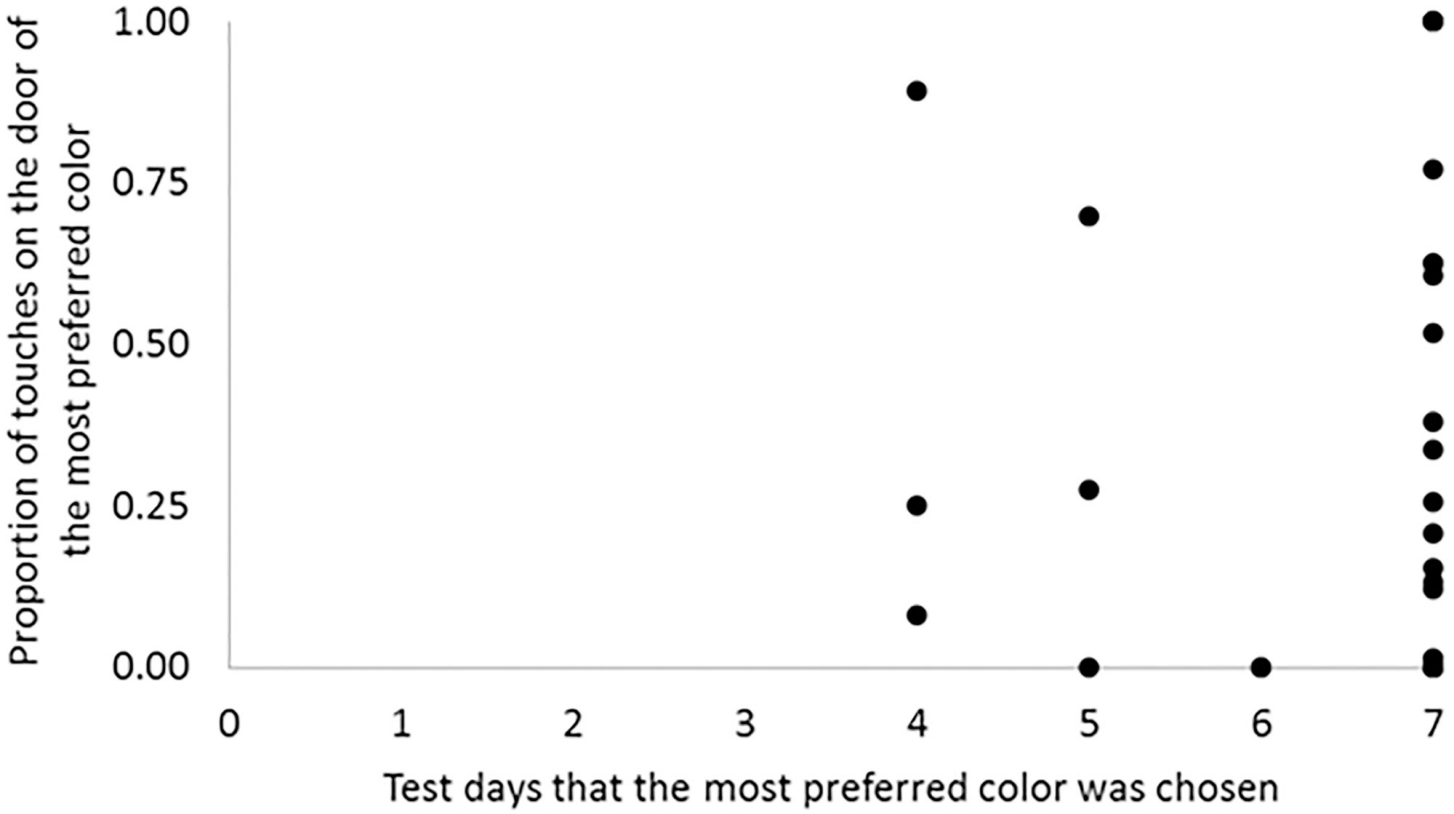
The number of test days that a fish chose its most preferred color did not affect its motivation response (the frequency of touches on the hinged, blocked doors) for that color. Individual data. Number of successive test days in which each fish preferred a same item contrasted with the motivation test. Note that most of the fish preferred a same item during all days of test (7 days), but the motivation varied greatly. N = 33 fish.

We also evaluated whether the motivation response could be affected by the amount of time the fish spent in a preferred color background. We found that 26 out of 33 fish showed a positive PI for the most preferred color over the entire 7 days of testing, with PI values increasing over time. A shorter period was observed for some fish: 3, 2, and 2 fish showed progressive PI values for the most preferred item for 6, 5 and 4 days, respectively. When we compared the touch intensity and the number of days showing positive and progressive PI values, there was no relation between these responses (Fig 5). This indicated that the association between motivation and PI did not depend on the number of days that the fish chose their preferred color.

## Discussion

In this study, we demonstrated that the motivation test corroborates the discrimination between preferred and non-preferred items by the PI, thus supporting preference and non-preferences as two levels of response with different biological relevance. This analysis provides a useful, reliable and easy tool to assess a key animal welfare issue: how to determine what animals prefer so that they can be housed and reared under better conditions (based on Dawkins’s theory [36]). Moreover, we confirmed individual variability in preference responses and, specifically to the Nile tilapia, blue background is reinforced as a color with welfare value, while yellow should not be considered.

Given that fish rejected the yellow color as their most preferred option and selected it as their most avoided option (Fig 2A), Nile tilapia showed that they prefer not to be on a yellow background color, at least within the context of the colors tested here. Such a response is also supported by the intensity of the preference, as the PIs for yellow were the lowest of the colors tested (Fig 2B). This makes sense because the fish contrasts with and is more visible against the yellow background—the lightest color in our tests. Thus, fish in the yellow compartment could be more stressed because they are more visible to potential predators; this assumption needs further investigation.

On the other hand, Luchiari *et al*. [33] demonstrated that Nile tilapia prefer yellow light. This different response to the color yellow may be because the preferences for different colors were evaluated by adding colored cellophane to the walls of transparent test aquaria, thus changing the color of the incident light, and did not evaluate different opaque background colors as tested here. In fact, preferences may vary depending on the different contextual conditions of the test [37]. However, other methodological differences might better explain these contradictory results. We evaluated preferences for 7 consecutive days following 3 days of adjustment to the test conditions, whereas Luchiari *et al*. [33] tested the preference responses immediately and twice a day over only 2 days. Maia & Volpato’s study [3] demonstrated that preference is not consistently detected in this context (Nile tilapia and color choices) before 3 days of test. This highlights the need to evaluate preference consistency over time and for a longer period than the 2 to 4 days over which these assays are typically scored.

In contrast, the color blue was determined to be one of the most preferred colors and also showed the highest PI values (Fig 2). Given that blue light reduces the stress response (cortisol [38] and ventilatory frequency [24]) and improves reproduction [28] in the Nile tilapia, we reinforce that the color blue might improve the welfare of this species and this supports our methodological approach.

With regard to the motivation response of the fish, Nile tilapia touched the hinged, blocked doors less frequently to access yellow and red colors. Thus, these fish were less motivated to access the color avoided in the preference test, yellow, and red. In fact, the color red impairs the growth of some fish species [39], including the Nile tilapia [27], possibly explaining why fish were less motivated to access the red background. Our approach is consistent with Duncan’s proposal [12] that evaluating an animal’s preferences for resources can be combined with testing for the motivation to access each specific resource, but the correspondence between PI and motivation tests suggest that the history-based method (PI) is a sufficiently accurate index to indicate animal preference.

Here, the differences between preference and motivation responses may have been a consequence of context. In other words, in the preference tests, fish could freely access any colored compartment, whereas in the motivation tests, fish were restricted to a white small chamber (Fig 1B). It is possible that the chamber could have stressed the fish, thus influencing the fish’s behavior on the motivation test. However, the size of the chamber was sufficiently large to minimize stress, as the fish were able to freely swim inside the chamber and remained in these conditions for only 5 min.

Although there were some differences between the preference and motivation responses for the colors, our findings also showed that the motivation to access items can be used to distinguish preferred from non-preferred items. The primary pattern (preference for blue and non-preference for yellow) was maintained between preference and motivation tests (Figs 2 and 3). More importantly, as the animals showed that they were more motivated to access their previously preferred items (identified by PI calculations), these findings demonstrate that the motivation tests empirically corroborate the PI proposed by Maia and Volpato [3] as a key tool to determine preferred and non-preferred options, which is of importance for the issue of animal welfare.

Single-preference fish exhibited the same avoidance response to yellow and a low motivation to access yellow and red colors. However, some variability was also observed in the responses (Figs 2A, 2B and 3A compared with Figs 2C, 2D and 3B). Single-preference fish exhibited a greater similarity between preference and motivation tests by avoiding and being less motivated to access both yellow and red colors (Figs 2C, 2D and 3B). These findings deserve more careful consideration; single-preference fish represent fish that are more decisive when given multiple-choice options, and this reinforces that motivational responses reflect the preferred options shown in the PI calculations.

Furthermore, Nile tilapia also expressed individual variability in color preferences. Fish were more motivated to access not only their individually most preferred color, but were more motivated also to reach all of their preferred colors compared with all of their non-preferred options (Fig 4). This fact could not be explained in terms of an effect of previous experience with a specific color, because we did not find correspondence of number of tests an item was preferred and motivation to reach such an item (Fig 5). Despite this, studies often evaluate preference (e.g. [1, 2]) and motivational responses (e.g. [4–8]) to determine the best conditions for a species irrespective of individual variability. However, our results highlight the need to consider individual variability whenever possible. These results are consistent with the significant individual variation in preferences demonstrated for other choice resources [17–20]. Thus, our findings support the PI [3] as a reliable and useful method to identify individually preferred and non-preferred options as two distinct biological responses.
