# Correction: Preference index supported by motivation tests in Nile tilapia

**DOI:** 10.1371/journal.pone.0192283

**Published:** 2018-01-29

**Authors:** Caroline Marques Maia, Gilson Luiz Volpato

The following information is missing from the Funding section: This work was supported by Conselho Nacional de Desenvolvimento Científico e Tecnológico (CNPq) as financial support through grant 307387/2013-5.
